# Apolar Polyisoprenoids Located in the Midplane of the Bilayer Regulate the Response of an Archaeal-Like Membrane to High Temperature and Pressure

**DOI:** 10.3389/fchem.2020.594039

**Published:** 2020-11-12

**Authors:** Josephine G. LoRicco, Marta Salvador-Castell, Bruno Demé, Judith Peters, Philippe M. Oger

**Affiliations:** ^1^Univ Lyon, INSA de Lyon, CNRS, MAP UMR 5240, Villeurbanne, France; ^2^Department of Large Scale Structures, Institut Laue-Langevin, Grenoble, France; ^3^Department of Spectroscopy, Université Grenoble Alpes, LiPhy, Grenoble, France

**Keywords:** archaea, archaeal lipids, high pressure, phase coexistence, membrane architecture, adaptation, membrane

## Abstract

Archaea are known to inhabit some of the most extreme environments on Earth. The ability of archaea possessing membrane bilayers to adapt to high temperature (>85°C) and high pressure (>1,000 bar) environments is proposed to be due to the presence of apolar polyisoprenoids at the midplane of the bilayer. In this work, we study the response of this novel membrane architecture to both high temperature and high hydrostatic pressure using neutron diffraction. A mixture of two diether, phytanyl chain lipids (DoPhPC and DoPhPE) and squalane was used to model this novel architecture. Diffraction data indicate that at high temperatures a stable coexistence of fluid lamellar phases exists within the membrane and that stable coexistence of these phases is also possible at high pressure. Increasing the amount of squalane in the membrane regulates the phase separation with respect to both temperature and pressure, and also leads to an increase in the lamellar repeat spacing. The ability of squalane to regulate the ultrastructure of an archaea-like membrane at high pressure and temperature supports the hypothesis that archaea can use apolar lipids as an adaptive mechanism to extreme conditions.

## Introduction

According to the Singer-Nicolson model, cell membranes are composed of a “mosaic” of proteins embedded in a fluid, lipid bilayer (Singer and Nicolson, 1972). Our understanding of cell membranes has developed further since then, for example we now recognize the importance of other lipid phases in addition to the fluid lamellar phase (bilayer phase), and that there can be substantial lateral heterogeneity within the membrane (Goñi, 2014). Membrane structural lipids, typically phospholipids, are diverse and display varied properties owing to the differences in both the lipid polar head groups and the hydrophobic tails. In aqueous solution, these lipids, driven by the hydrophobic effect, self-assemble into structures in which the hydrophilic heads can interact with the solution and the hydrophobic tails are excluded. The most biologically common phases, seen in familiar lipid bilayer structures, are lamellar phases in which the lipids assemble into flat sheets (zero membrane curvature) (Cullis et al., 1991; Perutková et al., 2009; Frolov et al., 2011; Goñi, 2014). However, the ability of membrane to form phases with non-zero curvature, such as cubic or inverted hexagonal phases, is also important and plays a role in many cellular processes such as membrane fission and fusion (Jouhet, 2013; McMahon and Boucrot, 2015; Jarsch et al., 2016). Membrane lipids can prefer different membrane curvatures based on their shape, and this preferred curvature can vary with environmental conditions such as pressure and temperature. The presence of diverse lipids within a membrane can even promote phase separation and domain formation within the membrane (Jouhet, 2013). Domains are laterally organized membrane regions with distinct lipid compositions and specialized functions such as interacting with specific proteins, or adopting a specific curvature (Tayebi et al., 2012; Arumugam and Bassereau, 2015; Marquardt et al., 2015). Such lateral membrane domains have been well-characterized in eukaryotic and bacterial cells (Baumgart et al., 2003; Heberle and Feigenson, 2011; Heberle et al., 2013; McCarthy et al., 2015; Schmid, 2017).

Life has been found at some of the most extreme conditions on Earth such as temperatures above 100°C and pressures up to 120 MPa (Yayanos et al., 1981; Takai et al., 2008; Zeng et al., 2009; Dalmasso et al., 2016; Siliakus et al., 2017). All aspects of these organisms must be specially adapted to tolerate such conditions. Cell membranes, in particular, are highly sensitive to pressure and temperature (Winter and Jeworrek, 2009; Oger and Jebbar, 2010; Brooks and Seddon, 2014). In order to maintain functionality of the membrane under extreme conditions cells adjust the composition of their membranes to cope with environmental changes, in a process known as homeoviscous adaptation (Sinensky, 1974). High temperature tends to increase membrane fluidity, permeability, and promote more negative membrane curvature whereas increasing pressure tends to have the opposite effects (Brooks, 2014). To compensate, bacteria and eukaryotes are known to regulate the length, saturation, and branching of the hydrophobic chains as well as the proportion of different polar headgroups (Jebbar et al., 2015; Siliakus et al., 2017). In archaea the mechanisms of adaptation are less well-understood, in part due to their unique membrane lipids. Archaeal lipids have methyl-branched phytanyl chains rather than straight chain fatty acids which are linked via ether rather than ester bonds to the glycerol backbone (De Rosa et al., 1986; Gambacorta et al., 1995). These lipids have higher temperature stability, and lowered proton permeability (Gliozzi et al., 1983; Yamauchi et al., 1993) compared with typical bacterial/eukaryotic lipids.

In addition to bilayer forming lipids, archaea are known to produce bipolar, tetra-ether lipids capable of forming lipid monolayers with high stability and low permeability (De Rosa et al., 1983; Elferink et al., 1994). Archaea have been shown to increase the quantity of tetra-ether lipids in the membrane in response to temperature (Matsuno et al., 2009; Cario et al., 2015). Even some bacteria have shown to produce membrane spanning tetraether or tetraester lipids in response to high-temperature conditions (Damsté et al., 2007; Schouten et al., 2007; Siliakus et al., 2017). Despite the link to high temperature adaption, tetra-ether lipids are not found solely in thermophiles but have also been observed in mesophilic archaea. In addition, not all organisms living at high temperatures produce large quantities, if any tetra-ether lipids (Tornabene and Langworthy, 1979; Hafenbradl et al., 1996; Sako et al., 1996; Sprott et al., 1997; Siliakus et al., 2017). Some insight into the ability of archaea with bilayer forming lipids to live at temperatures up to 100°C can be found in the study by Cario et al. (2015) on the piezo-hyperthermophilic archaeon, *Thermococcus barophilus*. The quantity and saturation of isoprenoid derivatives (such as lycopane, squalane) were shown to vary in response to temperature and pressure. In order to explain the ability of *T. barophilus* to live at high temperature in presence of bilayer forming lipids, it was hypothesized that apolar lipids sit at the midplane of the bilayer and provide enhanced stability (Cario et al., 2015). The ability of apolar lipids such as squalane to localize to the midplane of the lipid bilayer has been confirmed in model membranes of both bacterial-like and archaea-like lipids (Hauß et al., 2002; Salvador-Castell et al., 2020b). The presence of apolar molecules is capable of modulating membrane physicochemical properties, for example, the presence of squalane at the bilayer midplane has been shown to increase permeability to water and decrease proton permeability (Haines, 2001). Apolar molecules at the midplane have been shown to increase the tendency toward negative membrane curvature by reducing packing frustration (Salvador-Castell et al., 2020a,b) and to play a role in phase separation and domain formation (Gilmore et al., 2013; Salvador-Castell et al., 2020b). Such isoprenoid hydrocarbons are found in all archaea (Langworthy et al., 1982) suggesting this hypothesis could be extended to explain adaptation to high temperature and pressure in other archaea processing lipid bilayers.

In this work we studied the behavior of an archaeal-like bilayer with the proposed novel architecture composed of 1,2-di-O-phytanyl-*sn*-glycero-3-phosphocholine (DoPhPC) and 1,2-di-O-phytanyl-*sn*-glycero-3-phosphoethanolamine (DoPhPE) and 2,6,10,15,19,23-hexamethyltetracosane (squalane) under high temperature and high pressure conditions, mimicking the extreme conditions in which some archaea live. Using neutron diffraction, we are able to see the localization of squalane in the midplane bilayer, and to detect the presence of coexisting lamellar phases within the membrane. The structure of each phase as well as the phase coexistence were shown to vary in response to temperature and pressure. The response of the membrane ultrastructure to temperature and pressure could be modulated by varying the quantity of the apolar lipid squalane present in the membrane.

## Materials and Methods

### Chemicals

1,2-di-O-phytanyl-*sn*-glycero-3-phosphocholine (DoPhPC) and 1,2-di-O-phytanyl-*sn*-glycero-3-phosphoethanolamine (DoPhPE) were both purchased from Avanti Polar Lipids (Alabaster, USA) in the lyophilized form and utilized without further purification. The isoprenoid used, 2,6,10,15,19,23-hexamethyltetracosane (squalane) was bought from Sigma—Aldrich Co (Montana, USA) in its hydrogenated form and from CDN Isotopes (Pointe-Claire, Canada), in its deuterated form.

### Sample Preparation

Three milligram of DoPhPC:DoPhPE (9:1 molar) and the corresponding amount of either hydrogenated squalane (h-squalane) or deuterated squalane (d-squalane) were dissolved in chloroform:methanol (2:1) and were spread on a silicon wafer using the “rock and roll” method (Tristram-Nagle, 2007) and dried overnight under high vacuum. Next, the sample was hermetically sealed inside an aluminum sample holder containing a 1:1 ratio of H_2_O:D_2_O (50% D_2_O). The sample was left at 50°C for 48 h to allow for complete hydration.

### Neutron Diffraction

Neutron diffraction experiments were performed on D16 (Cristiglio et al., 2015) at the Institut Laue-Langevin (Grenoble, France). The incident wavelength was 4.52 Å. The accessible q-range was from 0.06 Å^−1^ to 0.51 Å^−1^. The H_2_O/D_2_O contrast was 50% D_2_O. 50% D_2_O was previously found to give both strong diffraction signal and good resolution between the first order peaks of the two lamellar phases. The temperature was carefully controlled by placing the sample holder in a cryostat. Samples were either measured in a high temperature (HT) sample holder or in a HT-high hydrostatic pressure (HHP) cell (Peters et al., 2018). Data obtained at ILL are identified by Salvador-Castell et al. (2019).

Data treatment was performed by LAMP (Richard et al., 1996) and Origin Pro (Version 2019, OriginLab Corperation, Northampton, MA, USA). The integrated intensities of the Bragg peaks were corrected according to the absorption and analyzed by a Gaussian function, as done previously (Salvador-Castell et al., 2020c). The angle (θ) of a Bragg peak is related to the scattering vector (*q*) by:

(1) $$\begin{array}{r} q=\frac{4\pi sin\left(\theta \right)}{\lambda } \end{array}$$

where λ is the wavelength. In cases where many orders of diffraction were visible, a linear fit of the form y = a + bx was performed on a plot of peak location (q) vs. diffraction order (h). The slope of the line (Δ*q*) was used to determine the d-spacing using the following equation:

(2) $$\begin{array}{r} d=\frac{2\pi }{\Delta q} \end{array}$$

In cases where only a single Bragg order was visible, the d-spacing was calculated using the first order peak and corrected based on the y-intercept determined for each sample under conditions where multiple peaks were present.

To locate squalane in the membrane we used the method described in Hauß et al. (2002, 2005) taking advantage of difference scattering density between hydrogen and deuterium. Neutron scattering length density (NSLD) profiles are constructed from the sum of the neutron scattering lengths per unit volume (Marquardt et al., 2015). The NSLD profiles were calculated from a discrete set of Fourier coefficients (*F*_*h*_) using the following equation (Katsaras, 1995):

(3) $$\begin{array}{r} \rho \left(z\right)=\frac{2}{d}\sum_{h\;=\;1}^{h_{max}}|F_{h}|v_{n}cos\left(\frac{2h\pi }{d}z\right) \end{array}$$

where *d* is the lamellar spacing of the bilayer in the z direction, $z\in \left[\frac{-d}{2},\frac{d}{2}\right]$, $|F_{h}|=\pm \sqrt{I_{h}Q_{z}}$, *Q*_*z*_ is the Lorentz correction factor equal to *q* for oriented bilayers, *I*_*h*_ is the integrated intensity of the hth Bragg peak and *v*_*n*_ is the phase of the structure factor. The assigned phases of structure factors 1 to 4 (–, +, +, –) were based on those previously determined for this [DoPhPC:DoPhPE(9:1) + squalane] (Salvador-Castell et al., 2020a).

## Results and Discussion

### Stable Phases Coexist Within Archaeal-Like Membrane at High Temperature and Pressure

Cario et al. (2015) proposed a novel membrane architecture to explain the stability of archaea lipid bilayers under high pressure and temperatures. In this novel membrane architecture, apolar lipids act as structural lipids, sitting at the midplane of the lipid bilayer and leading to enhanced membrane stability under extreme conditions (Oger and Cario, 2013; Cario et al., 2015; Salvador-Castell et al., 2020a). A synthetic archaeal-like membrane composed of a mixture of diphytanyl lipids (DoPhPC and DoPhPE in a 9 to 1 molar ratio) and the apolar lipid squalane was used to model this novel architecture. The chemical structures of these lipids are shown in Supplementary Figure 1. In order to probe how this membrane behaves under the extreme conditions of temperature and pressure faced by archaea, neutron diffraction was performed on oriented stacked bilayers at temperatures up to 85°C and pressures up to 1,000 bar. Experiments were performed with both hydrogenated squalane (h-squalane) and deuterated squalane (d-squalane) in order to take advantage of the differential neutron scattering between hydrogen and deuterium (Hauß et al., 2002, 2005). The neutron scattering length density (NSLD) for membranes containing h-squalane and d-squalane was plotted as a function of distance (Supplementary Figure 2). For convenience, 0 Å represents the midplane of the bilayer. These plots exhibit two characteristic maxima corresponding to the glycerol backbone and a minimal intensity near the terminal methyl groups. The spectra for the membrane containing h-squalane and d-squalane overlap fairly well except within the region corresponding to the midplane of the bilayer (−10 to 10 Å) where the sample containing d-squalane shows an excess of scattering density. This indicates that squalane is located in the midplane of the bilayer and is in agreement with the findings of Salvador-Castell et al. (2020b). In this work we see that this localization of squalane is seen in both Phase_LT_ and Phase_HT_ at low (20 bar) and high (1,000 bar) pressure.

**Figure 1:**
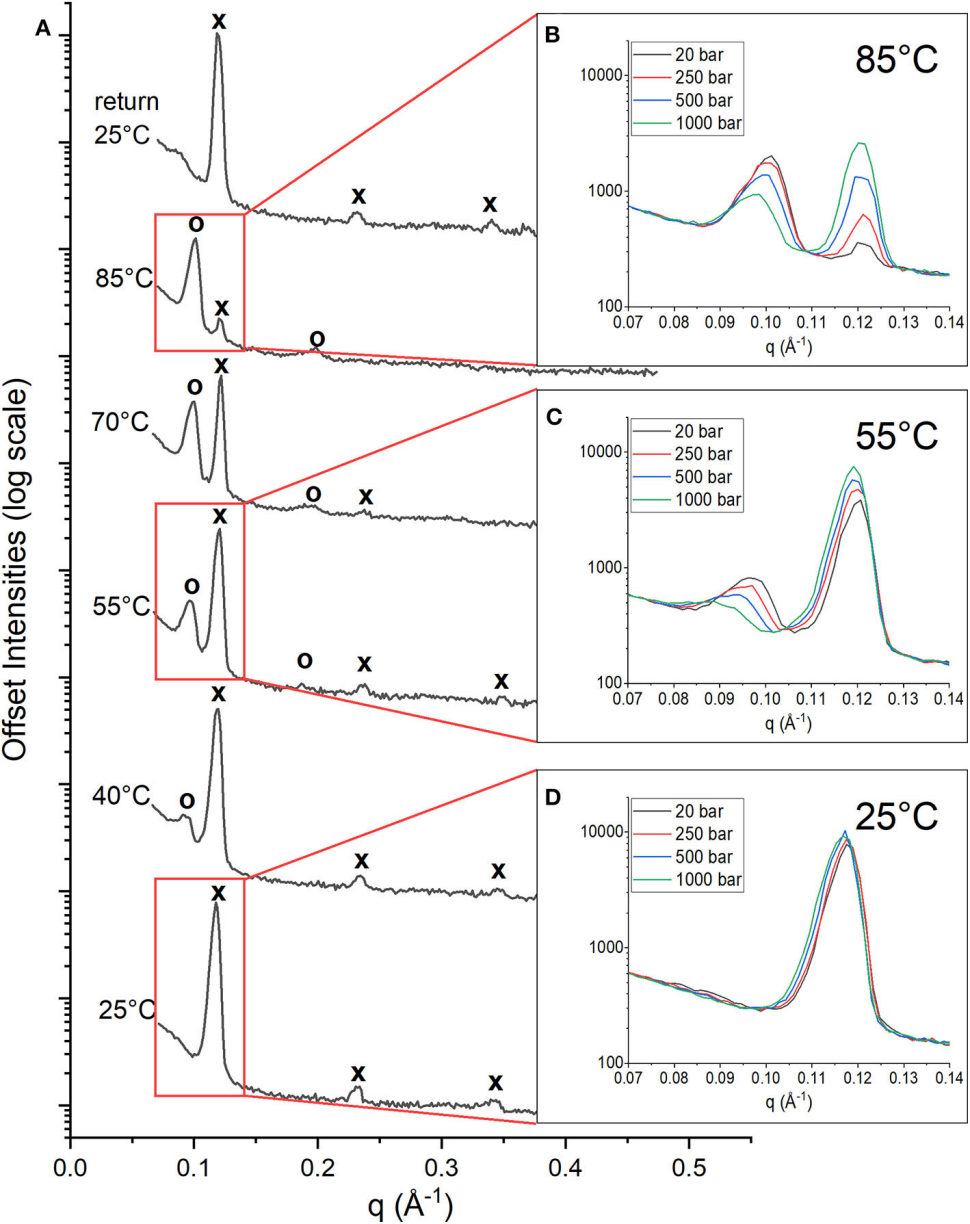
1D Neutron diffractograms of DoPhPC:DoPhPE (9:1) + 5 mol% h-squalane membrane. **(A)** Diffractograms at 20 bar and temperatures increasing from 25 to 85°C and after returning to 25°C. Diffraction peaks corresponding to Phase_LT_ are denoted with an “x” and peaks corresponding to Phase_HT_ are denoted with an “o.” First order diffraction peaks at 85°C **(B)**, 55°C **(C)**, and 25°C **(D)** measured at 20 bar (black), 250 bar (red), 500 bar (blue), and 1,000 bar (green).

The stacked multilayers were sufficiently ordered in the membrane to give rise to Bragg peaks in the diffraction data. Figure 1A shows the 1D neutron diffraction profiles for a membrane composed of DoPhPC:DoPhPE (9:1) + 5 mol% h-squalane at 20 bar (black spectra). The temperature increased from 25°C (bottom) to 85°C (top). At 25°C, there were three orders of diffraction for the sample at low pressure (20 bar). The first order diffraction peak was located at q = ~0.12 Å^−1^, the second order peak at q = ~0.23 Å^−1^, and the third order peak at q = ~0.34 Å^−1^. The location of peaks in the ratio of (1, 2, 3.) indicates a lamellar phase (Tyler et al., 2015). As the temperature increased, the peaks corresponding to the original phase were still present at similar values of q (marked with an “x”), although with diminished intensities. At higher temperatures (above 40°C) a second set of peaks appeared at lower values of q corresponding to a new phase (marked with an “o”). The first order diffraction peak of this new phase is located at q = ~0.09 Å^−1^ and the second order diffraction peak at q = ~0.18 Å^−1^. The spacing of the peaks indicates that this new phase is also lamellar. We will refer to the new phase as Phase_HT_ because it appeared at high temperatures (HT) compared to the original phase, which is also present at low temperatures (LT) and will therefore be referred to as Phase_LT_. Increasing temperature leads to an increase in the intensity of the Phase_HT_ peaks indicating that temperature stabilizes Phase_HT_, and leads to a decrease in the intensity of Phase_LT_ indicating that temperature destabilizes Phase_LT_. Upon returning the membrane to 25°C and 20 bar, the initial state, is restored indicating that this change in phase is fully reversible (Figure 1A).

At each temperature, the diffraction of the DoPhPC:DoPhPE (9:1) + 5 mol% h-squalane membrane was measured at 20, 250, 500, and 1,000 bar. The Figure 1 insets show the first order diffraction peaks, indicated by the red boxes, seen at pressures of 20 bar (black), 250 bar (red), 500 bar (blue) and 1,000 bar (green). At 85 and 55°C, two first order diffraction peaks are seen, representing the two phases. The area of the Phase_HT_ peak (found at lower q) decreases and the area of the Phase_LT_ peak (found at higher q) increases as a function of pressure (Figures 1B,C). This indicates that high pressure has a stabilizing effect on Phase_LT_ and a destabilizing effect on Phase_HT_. Although it is well-established that increasing pressure causes an effect similar to decreasing the temperature, our results show that even 1,000 bar is not enough to completely replace Phase_HT_ and return to the lipid organization present at lower temperatures. At 25°C, the membrane is exclusively in Phase_LT_. Again, there is a slight increase in the intensity of the Phase_LT_ peak with pressure indicating that pressure stabilizes Phase_LT_ (Figure 1D).

**Figure 2:**
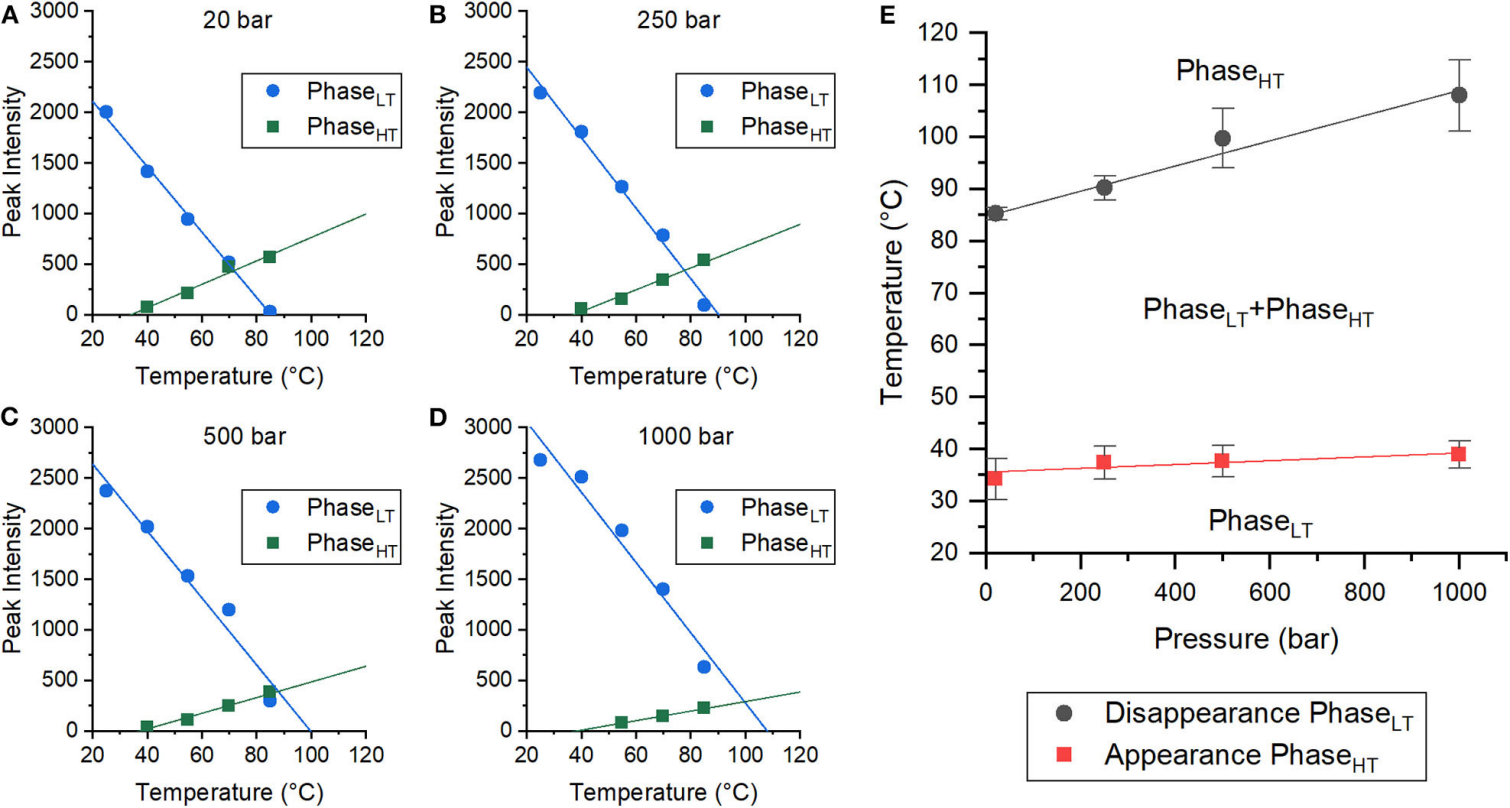
Coexistence of phases in DoPhPC:DoPhPE (9:1) membrane containing 5 mol% h-squalane. **(A–D)** Plots of peak intensities of Phase_LT_ (blue circles) and Phase_HT_ (green squares) as a function of temperature. Pressures were 20 bar **(A)**, 250 bar **(B)**, 500 bar **(C)**, and 1,000 bar **(D)**. Error bars are within symbols. **(E)** Pressure-Temperature diagram of the coexistence of Phase_LT_ and Phase_HT_. Error bars indicate standard errors.

Both pressure and temperature play an important role in the stability of each phase and their ability to coexist. In order to better quantify the temperature and pressure range at which these phases coexist, the change in the integrated peak intensities was monitored as a function of temperature at each pressure (20, 250, 500, and 1,000 bar). The changes in the peak intensities of the first order peaks of both Phase_LT_ and Phase_HT_ have a linear dependence on temperature (Figures 2A–D, Supplementary Figure 3). A linear fit could be used to extrapolate/interpolate when the peak intensity would be zero and thus to determine the temperature at which the corresponding phase was no longer present in the membrane for each pressure, as shown in Figure 2.

The integrated peak intensities of Phase_LT_ (blue) and Phase_HT_ (green) at 20 bar were analyzed as a function of temperature (Figure 2A). A linear fit of the integrated Phase_HT_ peak intensities determined that the peak intensity would equal zero at 34.3 ± 3.9°C, signifying that Phase_HT_ appears above this temperature. Below this temperature, the membrane is exclusively in Phase_LT_. A linear fit of the Phase_LT_ peak intensities determined that the peak intensity would reach zero at 85.3 ± 1.2°C. Above this temperature, Phase_LT_ would disappear and the membrane would be entirely in Phase_HT_. Between these two temperatures, both phases coexist. This analysis was repeated with the data taken at 250, 500, and 1,000 bar (Figures 2B–D), allowing the determination of a pressure-temperature diagram of the phase coexistence of Phase_LT_ and Phase_HT_ (Figure 2E). The temperature at which Phase_HT_ appears (T_HT_) increased with increasing pressure from 34.3 ± 3.9°C at 20 bar to 37.4 ± 3.2°C at 250 bar, 37.7 ± 3.0°C at 500 bar, and to 38.9 ± 2.6°C at 1,000 bar. The increase in T_HT_ with pressure was small (~5°C/kbar) and was not statistically significant. The temperature at which Phase_LT_ disappeared (T_LT_) also increased with increasing pressure from 85.3 ± 1.2°C at 20 bar, to 90.2 ± 2.3°C at 250 bar, 99.7 ± 5.7°C at 500 bar, and finally to 108.0 ± 6.9°C at 1,000 bar. The increase in T_LT_ as a function of pressure was much larger (~24°C/kbar) (Figure 2E). Here we demonstrate that although the phases are pressure sensitive, phase coexistence is possible at pressures up to, and presumably above, 1,000 bar. Based on the phase diagram, at high temperatures (> 85°C), the application of pressure is predicted to induce phase separation from solely Phase_HT_ at low pressure to a coexistence of Phase_HT_ and Phase_LT_ at high pressure and high temperature. Pressure induced phase separation is not a novel concept, as high pressure has been previously shown to be capable of inducing phase separation and domain formation (McCarthy et al., 2015). Our results suggest that this is also possible in membranes composed of archaeal lipids. It should be noted that the T_HT_ and T_LT_ reported here were calculated only as the sample being heated, and that the transition temperature upon cooling was not explicitly tracked. Membranes are known to exhibit hysteresis near phase transitions meaning that the transition temperatures upon heating and cooling are different. A similar effect has also been seen in membranes upon pressurization/depressurization (Trovaslet-Leroy et al., 2016).

**Figure 3:**
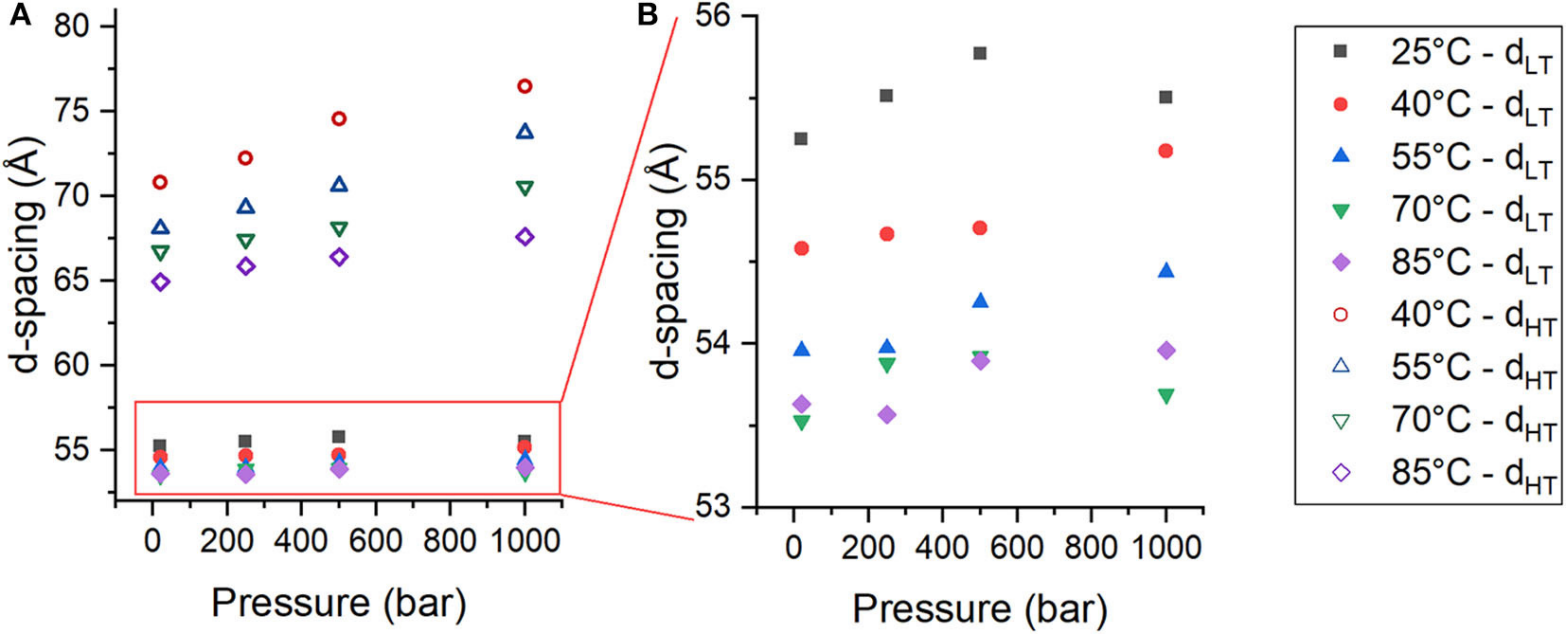
**(A,B)** Membrane d-spacing as a function of pressure for Phase_LT_ (solid symbols) and Phase_HT_ (open symbols) in DoPhPC:DoPhPE (9:1) membrane containing 5 mol% h-squalane. Temperature was 25°C (black squares), 40°C (red circles), 55°C (blue triangles), 70°C (green inverted triangles), or 85°C (purple diamonds). Error for d-spacing measurements is ± 0.5 Å.

From the changes in peak intensity, it could be determined that pressure destabilizes Phase_HT_ and stabilizes Phase_LT_. In addition to changes in the integrated intensities of the peaks, there were also changes in the location of the peaks with pressure that reflect changes in the lamellar repeat structure. The multilayer organization of the membranes makes it simple to determine the repeat spacing (d), which includes the thickness of the bilayer and its associated water layer (Nagle and Tristram-Nagle, 2000), from the location of the Bragg peaks of each phase. For a lamellar phase, the repeat spacing (d) can be calculated simply by Equation (3), where q is the location of the first order lamellar peak. The d-spacing of both Phase_LT_ (solid symbols) and Phase_HT_ (open symbols) increase as a function of pressure (Figure 3A). The d-spacing of Phase_LT_ with increasing pressure is shown again in Figure 3B using a different scale to better visualize the changes. Phase_LT_ exhibits either a small increase or negligible change in d-spacing with increasing pressure. Linear fitting of the data determined that the d-spacing of Phase_LT_ increases at a rate of 0.23 ± 0.32 Å/kbar at 25°C, 0.61 ± 0.13 Å/kbar at 40°C, 0.53 ± 0.11 at 55°C, 0.09 ± 0.30 at 70°C and 0.40 ± 0.16 at 85°C. An increase in membrane d-spacing with pressure has been observed previously (Winter and Jeworrek, 2009; Trapp et al., 2013; Brooks and Seddon, 2014). Increasing pressure provokes a lateral compression of membrane lipids and an increase in membrane thickness due to extension of the hydrocarbon chains. The d-spacing of Phase_HT_ also increases with pressure (Figure 3A). The d-spacing increased at a rate of 5.84 ± 0.81 Å/kbar at 40°C, 5.78 ± 0.19 Å/kbar at 55°C, 3.91 ± 0.36 Å/kbar at 70°C, and 2.62 ± 0.22 Å/kbar at 85°C. The changes in d-spacing for Phase_HT_ were almost an order of magnitude greater than that seen in Phase_LT_ showing that Phase_HT_ is much more sensitive to changes in pressure. It is interesting to note that the swelling of Phase_HT_ decreases with temperature.

The formation of a new phase, Phase_HT_, is most likely due to the presence of lipids with different preferred curvatures within the membrane as seen by Salvador-Castell et al. (2020b). The bilayer is made up of two phytanyl lipids with different polar head groups (DoPhPC and DoPhPE) and the apolar isoprenoid squalane. The phosphoethanolamine (PE) headgroup is small, so the lipid has a conical geometry and favors a negative curvature (favoring the formation of non-lamellar structure such as inverted hexagonal phases/cubic phases/inverted micelles). The phosphocholine (PC) headgroup is larger and the lipid has a cylindrical geometry which favors zero-curvature (favoring the formation of lamellar structures such as bilayers). The PC headgroup can stabilize the lamellar phase of PE lipids (Kates and Manson, 1984). The phase separation can be explained by partitioning of DoPhPC and DoPhPE into different phases based on preferred curvature which can be triggered, for example, by temperature which favors more negative membrane curvatures. The presence of squalane also promotes negative lipid curvature and was found to forward the formation of non-lamellar phases in a DoPhPC:DoPhPE (9:1) membrane (Salvador-Castell et al., 2020a,b). Increasing pressure, on the other hand, tends to increase the preferred membrane curvature (Shearman et al., 2006; Brooks, 2014). In our model membrane, phase separation was triggered by increasing temperature, which could be due to the differences in curvature between the lipids becoming too great to remain within the same phase. Increasing pressure hinders the phase separation and favors Phase_LT_, lending further support to the idea that the phase separation is driven by components of different curvature.

Increasing negative curvature favors a transition from a lamellar phase to a non-lamellar phase. Although no such phases were seen by neutron diffraction, non-lamellar phases in this membrane system have been detected by SAXS (Salvador-Castell et al., 2020a). Neutron scattering was performed on multi-stacks of bilayers on a silicon wafer, whereas for the SAXS the sample was not ordered on a substrate but rather was self-assembled in bulk. These differences in lipid preparation may explain why in one case the DoPhPC:DoPhPE (9:1) + 5 mol% squalane membrane separates into two lamellar phases and in the other case the membrane separates into a lamellar phase and an inverted hexagonal phase. The ordering of the lipids on a solid support for neutron diffraction may impede the formation of non-lamellar phases. For both phases seen by neutron diffraction, increasing pressure leads to an increase in the membrane d-spacing, however, the magnitude of the pressure-induced swelling was significantly different between the phases. The changes in Phase_LT_ are small, generally within the error of the measurements for the d-spacing. The changes in Phase_HT_ are quite large (2–6 Å/kbar). The pressure-induced swelling of the Phase_HT_ seen by neutron diffraction is unusually large for a lamellar phase which is typically <2 Å/kbar (Brooks, 2014). Interestingly, the magnitude of the pressure-induced swelling seen in Phase_HT_ is similar to that seen in the inverted hexagonal phase seen by SAXS (Salvador-Castell et al., 2020a). Supplementary Figure 4 illustrates how the membrane lattice parameter (equal to d for lamellar phase and 2/3 d for inverted hexagonal phase) increases as a function of pressure. The similarities in the response to pressure of Phase_HT_ and the inverted hexagonal phase seen in SAXS could be due to the partitioning of lipid headgroups and squalane in a similar manner.

### Squalane Regulates Membrane Ultrastructure Under High Temperature and Pressure

Archaea are thought to regulate their membranes response to extreme conditions with apolar lipids. For this reason, the neutron diffraction experiments were repeated with two additional concentrations of hydrogenated squalane, 2.5 and 10 mol% in order to determine what effect the amount of squalane would have on the membrane structure at high temperatures and pressures. Two coexisting phases are also present in membranes containing 2.5 or 10 mol% squalane at elevated temperatures (Supplementary Figures 5, 6).

**Figure 4:**
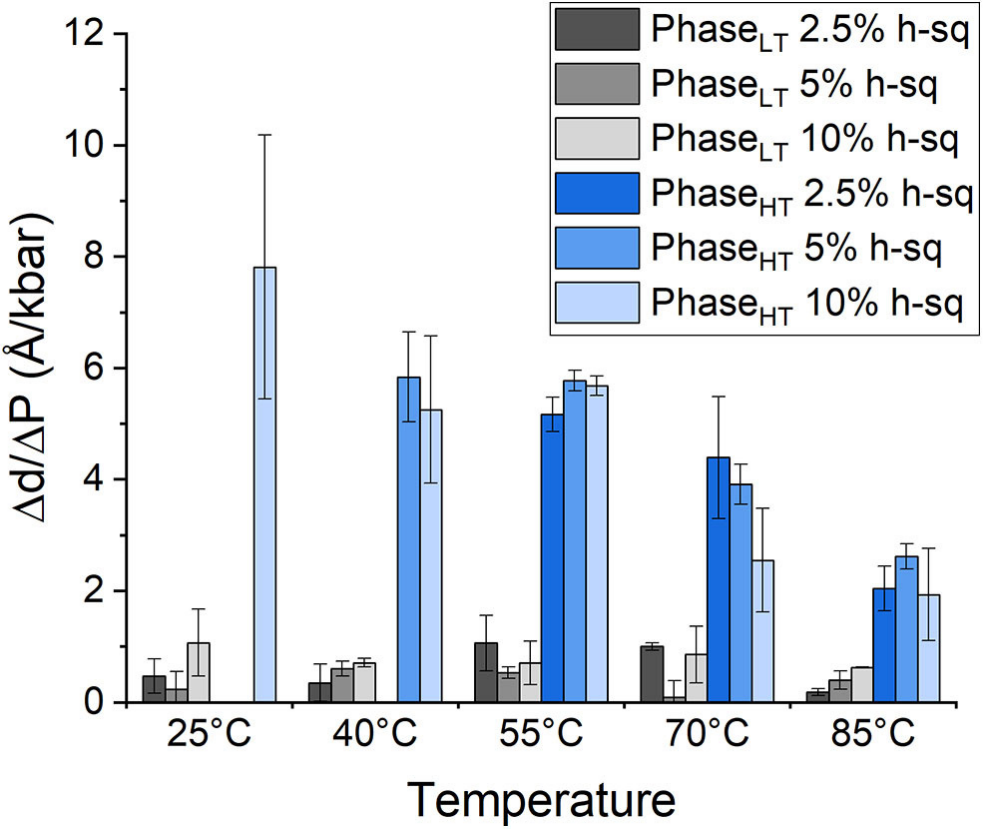
Pressure induced swelling of DoPhPC:DoPhPE (9:1) membrane containing different quantities of h-squalane. Swelling of Phase_LT_ containing 2.5 mol% (dark gray), 5 mol% (gray), or 10 mol% (light gray) h-squalane. Swelling of Phase_HT_ containing 2.5 mol% (dark blue), 5 mol% (blue), or 10 mol% (light blue) h-squalane.

The repeat spacing of Phase_LT_ and Phase_HT_ was calculated for the membranes containing 2.5, 5, and 10 mol% squalane and the values are displayed in Table 1. In all membranes, there is an increase in the membrane d-spacing with increasing pressure, and Phase_HT_ is again found to be the more pressure-sensitive phase (Figure 4). The pressure induced swelling of Phase_LT_ remains small (≤1Å/kbar) for all squalane concentrations and is not significantly different with changes in temperature. The pressure induced swelling of Phase_HT_ is much larger (2–8 Å/kbar) and the swelling of Phase_HT_ is temperature dependent. The swelling is significantly larger at low temperatures and smaller at high temperatures. Although the percentage of squalane does not change the swelling of Phase_HT_, the amount of squalane does play an important role in affecting the temperature range at which Phase_HT_ is present. At ≥55°C, Phase_HT_ is seen in membranes containing 2.5, 5, and 10 mol% squalane. At 40°C, Phase_HT_ is no longer capable of forming the membrane containing 2.5 mol% squalane and at 25°C, Phase_HT_ is only seen in the membrane containing 10 mol% squalane.

**Table 1:** Membrane d-spacing for Phase_LT_ (left table) and Phase_HT_ (right table) for DoPhPC:DoPhPE (9:1) membrane containing h-squalane.

**Phase_**LT**_**	**+2.5% squalane**	**+5% squalane**	**+10% squalane**	**Phase_**HT**_**	**+2.5% squalane**	**+5% squalane**	**+10% squalane**
25°C 20 bar	55.6 Å	55.2 Å	56.0 Å	25°C 20 bar	N.P.	N.P.	82.3 Å
25°C 250 bar	56.2 Å	55.5 Å	57.0 Å	25°C 250 bar	N.P.	N.P.	86.7 Å
25°C 500 bar	56.1 Å	55.8 Å	57.1 Å	25°C 500 bar	N.P.	N.P.	88.9 Å
25°C 1,000 bar	56.2 Å	55.5 Å	57.2 Å	25°C 1000bar	N.P.	N.P.	90.6 Å
40°C 20 bar	55.2 Å	54.6 Å	55.7 Å	40°C 20 bar	N.P.	70.8 Å	78.6 Å
40°C 250 bar	55.2 Å	54.7 Å	55.8 Å	40°C 250 bar	N.P.	72.2 Å	79.8 Å
40°C 500 bar	55.0 Å	54.7 Å	56.0 Å	40°C 500 bar	N.P.	74.6 Å	82.6 Å
40°C 1,000 bar	55.6 Å	55.2 Å	56.4 Å	40°C 1,000 bar	N.P.	76.5 Å	83.6 Å
55°C 20 bar	54.1 Å	54.0 Å	55.3 Å	55°C 20 bar	67.1 Å	68.1 Å	74.7 Å
55°C 250 bar	54.8 Å	54.0 Å	55.7 Å	55°C 250 bar	68.5 Å	69.3 Å	75.9 Å
55°C 500 bar	55.2 Å	54.3 Å	55.3 Å	55°C 500 bar	69.4 Å	70.6 Å	77.3 Å
55°C 1,000 bar	55.2 Å	54.4 Å	56.1 Å	55°C 1,000 bar	72.2 Å	73.7 Å	80.0 Å
70°C 20 bar	53.8 Å	53.5 Å	54.4 Å	70°C 20 bar	65.7 Å	66.8 Å	72.4 Å
70°C 250 bar	54.0 Å	53.9 Å	55.3 Å	70°C 250 bar	65.7 Å	67.5 Å	74.3 Å
70°C 500 bar	54.2 Å	53.9 Å	55.2 Å	70°C 500 bar	67.7 Å	68.2 Å	74.1 Å
70°C 1,000 bar	54.8 Å	53.7 Å	55.2 Å	70°C 1,000 bar	70.1 Å	70.6 Å	75.3 Å
85°C 20 bar	53.5 Å	53.6 Å	N.P.	85°C 20 bar	64.9 Å	65.0 Å	74.1 Å
85°C 250 bar	53.5 Å	53.6 Å	54.3 Å	85°C 250 bar	65.4 Å	65.9 Å	75.4 Å
85°C 500 bar	53.6 Å	53.9 Å	54.5 Å	85°C 500 bar	65.9 Å	66.4 Å	74.8 Å
85°C 1,000 bar	53.7 Å	54.0 Å	54.8 Å	85°C 1,000 bar	67.3 Å	67.6 Å	76.3 Å

*N.P., not present. Error is ± 0.5 Å*.

The temperatures at which Phase_HT_ appears for a membrane containing either 2.5 or 10 mol% squalane are determined from a linear fit of integrated peak intensity vs. temperature (Supplementary Figure 3). As seen previously with the membrane containing 5 mol% squalane, pressure leads to an increase in T_HT_ and T_LT_ (Supplementary Figure 7). Increasing the quantity of squalane promotes the formation of Phase_HT_ at lower temperatures. For example, at 20 bar Phase_HT_ appears at 44.7 ± 4.0°C for the membrane containing 2.5 mol% squalane, 34.3 ± 3.9°C for the membrane containing 5 mol% squalane and 23.9 ± 3.4°C for the membrane containing 10 mol% squalane. The concentration of squalane has a similar effect on T_HT_ at elevated pressures (Table 2).

**Table 2:** Temperature at which Phase_HT_ appears in DoPhPC:DoPhPE (9:1) membrane containing h-squalane.

**Pressure**	**2.5% squalane**	**5% squalane**	**10% squalane**
20 bar	44.7 ± 4.0°C	34.3 ± 3.9°C	23.9 ± 3.4°C
250 bar	49.3 ± 1.5°C	37.4 ± 3.2°C	28.8 ± 3.3°C
500 bar	49.2 ± 0.1°C	37.7 ± 3.0°C	31.8 ± 1.6°C
1,000 bar	50.8 ± 1.2°C	38.9 ± 2.6°C	32.2 ± 2.6°C

This agrees with previous findings that squalane promotes negative membrane curvature, favoring the formation of Phase_HT_ and phase separation at lower temperatures (Salvador-Castell et al., 2020a,b). Here we show that squalane is also capable of promoting the formation of Phase_HT_ at high pressures. Previously at ambient pressure, it was shown that the phase coexistence and lateral organization within a DoPhPC:DoPhPE membrane was squalane dependent leading to the hypothesis that squalane may promote the formation of membrane domains (Salvador-Castell et al., 2020b). That would mean that the ability of squalane to favor phase separation at high pressures, may indicate that squalane could also promote domain formation in archaea that live at such high-pressure conditions.

In addition to regulating the phase coexistence within the membrane, increasing the amount of squalane in the membrane also affects the d-spacing. The d-spacing of the membrane is typically higher when the membrane contains a higher quantity of squalane for Phase_HT_ (Table 1, Supplementary Figure 8). For example, in Phase_HT_ at 20 bar/55°C the d-spacing is 67.1 Å when the membrane contains 2.5 mol% squalane compared with 68.1 Å when the membrane contains 5 mol% squalane and 74.7 Å when the membrane contains 10 mol% squalane. In this experiment, we did not see a significant change in the d-spacing of Phase_LT_ with increasing squalane concentration which was seen previously, at ambient pressure and temperature (Salvador-Castell et al., 2020b). The increase in d-spacing with increasing squalane for Phase_LT_ was only seen up to 5 mol% squalane at which point the phase is thought to reach saturation. It is conceivable that the amount of squalane required to reach saturation within membrane could change with pressure and temperature and this may be one of the reason we did not see any change in the d-spacing of Phase_LT_ with increasing squalane in this experiment.

At temperatures at which Phase_HT_ was present, Phase_HT_ was always found to increase in d-spacing with increasing squalane. Previously it was shown that increasing the percentage of squalane leads to an increase in the d-spacing in Phase_LT_ due to an increase in the hydrophobic core thickness (Salvador-Castell et al., 2020b). The localization of squalane to the midplane of the bilayer is confirmed for Phase_HT_ as well as Phase_LT_ (Supplementary Figure 2). This could indicate that the increase in the d-spacing for Phase_HT_ is also due to an increase in hydrophobic core thickness although this cannot be directly confirmed from this data. Increasing squalane promotes changes in membrane structure of both phases, even at high pressures and temperatures, indicating that squalane could act as a membrane regulator under the extreme conditions at which many archaea live.

## Conclusions

It was proposed by Cario et al. (2015) that apolar lipids, such as squalane, sit at the midplane of the lipid bilayer, and provide a means of enhancing the stability of archaeal membrane bilayers under extreme conditions. This localization of apolar lipids to the midplane of an archaeal-like bilayer was then confirmed by the work of Salvador-Castell et al. (2020b). The aims of this study were to determine how an archaeal-like membrane with this novel membrane architecture behaves in response to the high temperatures and high hydrostatic pressures and to determine how the quantity of apolar lipid present in the membrane modulates this behavior. To model the proposed membrane architecture in which apolar molecules sit in the midplane of the bilayer, we used an artificial archaea-like membrane composed of DoPhPC, DoPhPE, and squalane. The use of neutron diffraction allowed us to confirm the localization of the apolar lipid squalane and to further study the coexistence of two distinct lamellar phases previously reported by Salvador-Castell et al. (2020b). The phase separation within the membrane is most likely due to the partitioning of lipids (DoPhPC/DoPhPE) with different preferred curvatures and the presence of the apolar molecule (squalane). The two phases are capable of coexisting at a wide range of temperatures and pressure. High temperature favors the formation of a swollen lamellar phase (Phase_HT_) and high pressure favors a thinner lamellar phase (Phase_LT_). The temperature and pressure range of the phase coexistence is regulated by the percentage of squalane present in the membrane. Increasing the percentage of squalane favors the formation of Phase_HT_ at lower temperatures, for pressures up to 1,000 bar. Coexistence and lateral organization of these phases was seen previously at ambient pressure (Salvador-Castell et al., 2020b). Here we have shown that phase coexistence is also seen at high pressure which could indicate the possibility of domain formation in archaea living at high pressure in addition to high temperature. Increasing the amount of squalane also leads to an increase in the membrane lamellar repeat spacing for Phase_HT_. The ability of squalane to modify the membrane ultrastructure at both high pressure and temperature supports the hypothesis that apolar lipids play a role in adaptation of archaea to extreme conditions.

## Data Availability Statement

The datasets generated for this study can be found in online repositories. The names of the repository/repositories and accession number(s) can be found at: http://dx.doi.org/10.5291/ILL-DATA.8-02-852.

## Author Contributions

PO, MS-C, and JP conceived the project. MS-C, BD, JP, and PO carried out the experiments. JL performed the data analysis and wrote the initial draft. All authors contributed to the final manuscript.

## Conflict of Interest

The authors declare that the research was conducted in the absence of any commercial or financial relationships that could be construed as a potential conflict of interest.

## References

[B1] Arumugam S. Bassereau P. (2015). Membrane nanodomains: contribution of curvature and interaction with proteins and cytoskeleton. Essays Biochem. 57, 109–119. 10.1042/bse0570109 25658348

[B2] Baumgart T. Hess S. T. Webb W. W. (2003). Imaging coexisting fluid domains in biomembrane models coupling curvature and line tension. Nature 425, 821–824. 10.1038/nature02013 14574408

[B3] Brooks N. J. (2014). Pressure effects on lipids and bio-membrane assemblies. IUCrJ 1, 470–477. 10.1107/S2052252514019551 25485127 PMC4224465

[B4] Brooks N. J. Seddon J. M. (2014). High pressure x-ray studies of lipid membranes and lipid phase transitions. Zeitschr. Phys. Chem. 228, 987–1004. 10.1515/zpch-2014-0602

[B5] Cario A. Grossi V. Schaeffer P. Oger P. M. (2015). Membrane homeoviscous adaptation in the piezo-hyperthermophilic archaeon *Thermococcus barophilus*. Front. Microbiol. 6:1152. 10.3389/fmicb.2015.01152 26539180 PMC4612709

[B6] Cristiglio V. Giroud B. Didier L. Demé B. (2015). D16 is back to business: more neutrons, more space, more fun. Neutron News 26, 22–24. 10.1080/10448632.2015.1057051

[B7] Cullis P. R. Tilcock C. P. Hope M. J. (1991). “Lipid polymorphism,” in Membrane Fusion, eds J. Wilschut and D. Hoekstra (New York, NY: CRC Press), 35–64.

[B8] Dalmasso C. Oger P. Courtine D. Georges M. Takai K. Maignien L.. (2016). Complete genome sequence of the hyperthermophilic and piezophilic archeon *Thermococcus piezophilus* CDGST, able to grow under extreme hydrostatic pressures. Genome Announc. 4:e00610-16. 10.1128/genomeA.00610-16 27417831 PMC4945791

[B9] Damsté J. S. S. Rijpstra W. I. C. Hopmans E. C. Schouten S. Balk M. Stams A. J. M. (2007). Structural characterization of diabolic acid-based tetraester, tetraether and mixed ether/ester, membrane-spanning lipids of bacteria from the order thermotogales. Arch. Microbiol. 188, 629–641. 10.1007/s00203-007-0284-z 17643227 PMC2111041

[B10] De Rosa M. Gambacorta A. Gliozzi A. (1986). Structure, biosynthesis, and physicochemical properties of archaebacterial lipids. Microbiol. Rev. 50, 70–80. 10.1128/MMBR.50.1.70-80.1986 3083222 PMC373054

[B11] De Rosa M. Gambacorta A. Nicolaus B. (1983). A new type of cell membrane, in thermophilic archaebacteria, based on bipolar ether lipids. J. Memb. Sci. 16, 287–294. 10.1016/S0376-7388(00)81316-2

[B12] Elferink M. G. L. De Wit J. G. Driessen A. J. M. Konings W. N. (1994). Stability and proton-permeability of liposomes composed of archaeal tetraether lipids. Biochim. Biophys. Acta 1193, 247–254. 10.1016/0005-2736(94)90160-0 8054346

[B13] Frolov V. A. Shnyrova A. V. Zimmerberg J. (2011). Lipid polymorphisms and membrane shape. Cold Spring Harb. Perspect. Biol. 3:a004747. 10.1101/cshperspect.a004747 21646378 PMC3220359

[B14] Gambacorta A. Gliozzi A. De Rosa M. (1995). Archaeal lipids and their biotechnological applications. World J. Microbiol. Biotechnol. 11, 115–131. 10.1007/BF00339140 24414415

[B15] Gilmore S. F. Yao A. I. Tietel Z. Kind T. Facciotti M. T. Parikh A. N. (2013). Role of squalene in the organization of monolayers derived from lipid extracts of *Halobacterium salinarum*. Langmuir 29, 7922–7930. 10.1021/la401412t 23713788 PMC4438081

[B16] Gliozzi A. Paoli Mario Rosa G. D. E. Gambacorta A. (1983). Effect of isoprenoid cyclization on the transition temperature of lipids in thermophilic archaebacteria. Biochem. Biophys. Acta 735, 234–242. 10.1016/0005-2736(83)90298-5

[B17] Goñi F. M. (2014). The basic structure and dynamics of cell membranes: an update of the Singer-Nicolson model. Biochim. Biophys. Acta Biomembr. 1838, 1467–1476. 10.1016/j.bbamem.2014.01.006 24440423

[B18] Hafenbradl D. Keller M. Stetter K. O. (1996). Lipid analysis of *Methanopyrus kandleri*. FEMS Microbiol. Lett. 136, 199–202. 10.1111/j.1574-6968.1996.tb08049.x

[B19] Haines T. H. (2001). Do sterols reduce proton and sodium leaks through lipid bilayers? Prog. Lipid Res. 40, 299–324. 10.1016/S0163-7827(01)00009-1 11412894

[B20] Hauß T. Dante S. Dencher N. A. Haines T. H. (2002). Squalane is in the midplane of the lipid bilayer: implications for its function as a proton permeability barrier. Biochem. Biophys. Acta 1556, 149–154. 10.1016/S0005-2728(02)00346-8 12460672

[B21] Hauß T. Dante S. Haines T. H. Dencher N. A. (2005). Localization of coenzyme Q10 in the center of a deuterated lipid membrane by neutron diffraction. Biochim. Biophys. Acta Bioenerg. 1710, 57–62. 10.1016/j.bbabio.2005.08.007 16199002

[B22] Heberle F. A. Feigenson G. W. (2011). Phase separation in lipid membranes. Cold Spring Harb. Perspect. Biol. 3, 1–13. 10.1101/cshperspect.a004630 21441593 PMC3062215

[B23] Heberle F. A. Petruzi1‘1elo R. S. Pan J. Drazba P. Kučerka N. Standaert R. F.. (2013). Bilayer thickness mismatch controls domain size in model membranes. J. Am. Chem. Soc. 135, 6853–6859. 10.1021/ja3113615 23391155

[B24] Jarsch I. K. Daste F. Gallop J. L. (2016). Membrane curvature in cell biology: an integration of molecular mechanisms. J. Cell Biol. 214, 375–387. 10.1083/jcb.201604003 27528656 PMC4987295

[B25] Jebbar M. Franzetti B. Girard E. Oger P. (2015). Microbial diversity and adaptation to high hydrostatic pressure in deep-sea hydrothermal vents prokaryotes. Extremophiles 19, 721–740. 10.1007/s00792-015-0760-3 26101015

[B26] Jouhet J. (2013). Importance of the hexagonal lipid phase in biological membrane organization. Front. Plant Sci. 4:494. 10.3389/fpls.2013.00494 24348497 PMC3848315

[B27] Kates M. Manson L. A. (1984). Membrane Fluidity. New York, NY: Plenum Press.

[B28] Katsaras J. (1995). X-ray diffraction studies of oriented lipid bilayers. Biochem. Cell Biol. 73, 209–218. 10.1139/o95-025 8829365

[B29] Langworthy T. A. Tornabene T. G. Holzer G. (1982). Lipids of Archaebacteria. Zent. Bakteriol. Angew. Okol. Microbiol. Abt. L. Orig. C. Hyg. 3, 228–244.

[B30] Marquardt D. Heberle F. A. Nickels J. D. Pabst G. Katsaras J. (2015). On scattered waves and lipid domains: detecting membrane rafts with X-rays and neutrons. Soft Matter. 11, 9055–9072. 10.1039/C5SM01807B 26428538 PMC4719199

[B31] Matsuno Y. Sugai A. Higashibata H. Fukuda W. Ueda K. Uda I.. (2009). Effect of growth temperature and growth phase on the lipid composition of the archaeal membrane from Thermococcus kodakaraensis. Biosci. Biotechnol. Biochem. 73, 104–108. 10.1271/bbb.80520 19129645

[B32] McCarthy N. L. C. Ces O. Law R. V. Seddon J. M. Brooks N. J. (2015). Separation of liquid domains in model membranes induced with high hydrostatic pressure. Chem. Commun. 51, 8675–8678. 10.1039/C5CC02134K 25907808

[B33] McMahon H. T. Boucrot E. (2015). Membrane curvature at a glance. J. Cell Sci. 128, 1065–1070. 10.1242/jcs.114454 25774051 PMC4359918

[B34] Nagle J. F. Tristram-Nagle S. (2000). Structure of lipid bilayers. Biochim. Biophys. Acta 1469, 159–195. 10.1016/S0304-4157(00)00016-2 11063882 PMC2747654

[B35] Oger P. M. Cario A. (2013). Adaptation of the membrane in archaea. Biophys. Chem. 183, 42–56. 10.1016/j.bpc.2013.06.020 23915818

[B36] Oger P. M. Jebbar M. (2010). The many ways of coping with pressure. Res. Microbiol. 161, 799–809. 10.1016/j.resmic.2010.09.017 21035541

[B37] Perutková Š. Daniel M. Dolinar G. Rappolt M. Kralj-Iglič V. Iglič A. (2009). Stability of the inverted hexagonal phase, Adv. Planar Lipid Bilayers Liposomes, 9, 237–278. 10.1016/S1554-4516(09)09009-7

[B38] Peters J. Golub M. Demé B. Gonthier J. Maurice J. Payre C. (2018). New pressure cells for membrane layers and systems in solutions up to 100°C. J. Neutron Res. 20, 1–10. 10.3233/JNR-180055

[B39] Richard D. Ferrand M. Kearley G. J. (1996). Analysis and visualisation of neutron-scattering data. J. Neutron Res. 4, 33–39. 10.1080/10238169608200065

[B40] Sako Y. Nomura N. Uchida A. Ishida Y. Morii H. Koga Y.. (1996). *Aeropyrum pernix* gen. nov., sp. nov., a novel aerobic hyperthermophilic archaeon growing at temperatures up to 100°C. Int. J. Syst. Bacteriol. 46, 1070–1077. 10.1099/00207713-46-4-1070 8863437

[B41] Salvador-Castell M. Brooks N. J. Peters J. Oger P. (2020a). Induction of non-lamellar phases in archaeal lipids at high temperature and high hydrostatic pressure by apolar polyisoprenoids. Biochim. Biophys. Acta Biomembr. 1862:183130. 10.1016/j.bbamem.2019.183130 31734311

[B42] Salvador-Castell M. Déme B. Misuraca L. Oger P. Peters J. (2019). Novel High-Pressure/High-Temperature Phases in Archaeal Membranes. Institut Laue-Langevin (ILL). 10.5291/ILL-DATA.8-02-852

[B43] Salvador-Castell M. Demé B. Oger P. Peters J. (2020b). Lipid phase separation induced by the apolar polyisoprenoid squalane demonstrates its role in membrane domain formation in archaeal membranes. Langmuir 36, 7375–7382. 10.1021/acs.langmuir.0c00901 32515591

[B44] Salvador-Castell M. Demé B. Oger P. Peters J. (2020c). Structural characterization of an archaeal lipid bilayer as a function of hydration and temperature. Int. J. Mol. Sci. 21:1816. 10.3390/ijms21051816 32155764 PMC7084678

[B45] Schmid F. (2017). Physical mechanisms of micro- and nanodomain formation in multicomponent lipid membranes. Biochim. Biophys. Acta 1859, 509–528. 10.1016/j.bbamem.2016.10.021 27823927

[B46] Schouten S. Van Der Meer M. T. J. Hopmans E. C. Rijpstra W. I. C. Reysenbach A. L. Ward D. M.. (2007). Archaeal and bacterial glycerol dialkyl glycerol tetraether lipids in hot springs of Yellowstone National Park. Appl. Environ. Microbiol. 73, 6181–6191. 10.1128/AEM.00630-07 17693566 PMC2074994

[B47] Shearman G. C. Ces O. Templer R. H. Seddon J. M. (2006). Inverse lyotropic phases of lipids and membrane curvature. J. Phys. Condens. Matter 18:S1105–24. 10.1088/0953-8984/18/28/S01 21690832

[B48] Siliakus M. F. van der Oost J. Kengen S. W. M. (2017). Adaptations of archaeal and bacterial membranes to variations in temperature, pH and pressure. Extremophiles 21, 651–670. 10.1007/s00792-017-0939-x 28508135 PMC5487899

[B49] Sinensky M. (1974). Homeoviscous adaptation: a homeostatic process that regulates the viscosity of membrane lipids in *Escherichia coli*. Proc. Natl. Acad. Sci. U.S.A. 71, 522–525. 10.1073/pnas.71.2.522 4360948 PMC388039

[B50] Singer S. J. Nicolson G. L. (1972). The fluid mosaic model of the structure of cell membranes. Science 175, 720–731. 10.1126/science.175.4023.720 4333397

[B51] Sprott G. D. Agnew B. J. Patel G. B. (1997). Structural features of ether lipids in the archaeobacterial thermophiles pyrococcus furiosus, *Methanopyrus kandleri, Methanothermus fervidus*, and *Sulfolobus acidocaldarius*. Can. J. Microbiol. 43, 467–476. 10.1139/m97-066

[B52] Takai K. Nakamura K. Toki T. Tsunogai U. Miyazaki M. Miyazaki J.. (2008). Cell proliferation at 122°C and isotopically heavy CH4 production by a hyperthermophilic methanogen under high-pressure cultivation. Proc. Natl. Acad. Sci. U.S.A. 105, 10949–10954. 10.1073/pnas.0712334105 18664583 PMC2490668

[B53] Tayebi L. Ma Y. Vashaee D. Chen G. Sinha S. K. Parikh A. N. (2012). Long-range interlayer alignment of intralayer domains in stacked lipid bilayers. Nat. Mater. 11, 1074–1080. 10.1038/nmat3451 23085566

[B54] Tornabene T. G. Langworthy T. A. (1979). Diphytanyl and dibiphytanyl glycerol ether lipids of methanogenic archaebacteria. Science 203, 51–53. 10.1126/science.758677 758677

[B55] Trapp M. Marion J. Tehei M. Demé B. Gutberlet T. Peters J. (2013). High hydrostatic pressure effects investigated by neutron scattering on lipid multilamellar vesicles. Phys. Chem. Chem. Phys. 15, 20951–20956. 10.1039/c3cp52762j 24201561

[B56] Tristram-Nagle S. A. (2007). Preparation of oriented, fully hydrated lipid samples for structure determination using X-ray scattering. Methods Mol. Biol. 400, 63–75. 10.1007/978-1-59745-519-0_5 17951727 PMC2697614

[B57] Trovaslet-Leroy M. Martinez N. Marion J. Peters J. (2016). “Hautes pressions et dynamique des systems bilogiques,” in Mesures en Conditions Extrêmes, eds Y. Le Godec, H. Cardon, T. Hammouda, and Y. Morizet (Paris: Éditions des Archives Contemporaines), 51–64.

[B58] Tyler A. I. I. Law R. V. Seddon J. M. (2015). “X-Ray diffraction of lipid model membranes,” in Methods in Membrane Lipids, ed D. M. Owen (New York, NY: Humana Press), 199–225. 10.1007/978-1-4939-1752-5_16 25331138

[B59] Winter R. Jeworrek C. (2009). Effect of pressure on membranes. Soft Matter. 5, 3157–3173. 10.1039/b901690b

[B60] Yamauchi K. Doi K. Yoshida Y. Kinoshita M. (1993). Archaebacterial lipids: highly proton-impermeable membranes from 1,2-diphytanyl-sn-glycero-3-phosphocholine. Biochim. Biophys. Acta 1146, 178–182. 10.1016/0005-2736(93)90353-2 8383997

[B61] Yayanos A. A. Dietz A. S. Van Boxtel R. (1981). Obligately barophilic bacterium from the mariana trench. Proc. Natl. Acad. Sci. U.S.A. 78, 5212–5215. 10.1073/pnas.78.8.5212 6946468 PMC320377

[B62] Zeng X. Birrien J. L. Fouquet Y. Cherkashov G. Jebbar M. Querellou J.. (2009). Pyrococcus CH1, an obligate piezophilic hyperthermophile: extending the upper pressure-temperature limits for life. ISME J. 3, 873–876. 10.1038/ismej.2009.21 19295639

