# Supplementary material for: Apolar Polyisoprenoids Located in the Midplane of the Bilayer Regulate the Response of an Archaeal-Like Membrane to High Temperature and Pressure
Source: Front Chem. 2020 Nov 12;8:594039. doi: 10.3389/fchem.2020.594039 (PMC7689154; doi:10.3389/fchem.2020.594039)
Supplement: Supplementary file 1 [file Data_Sheet_1.PDF]

Supplementary Material

1.1 Supplementary Figures

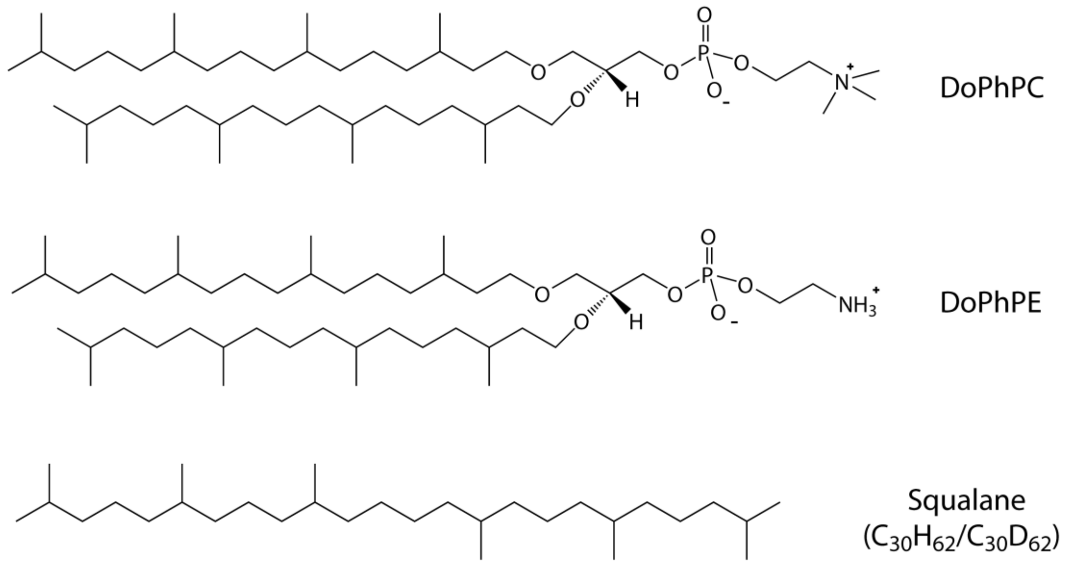

Supplementary Figure 1. Chemical structure of DoPhPC, DoPhPE, and squalane used in this study.

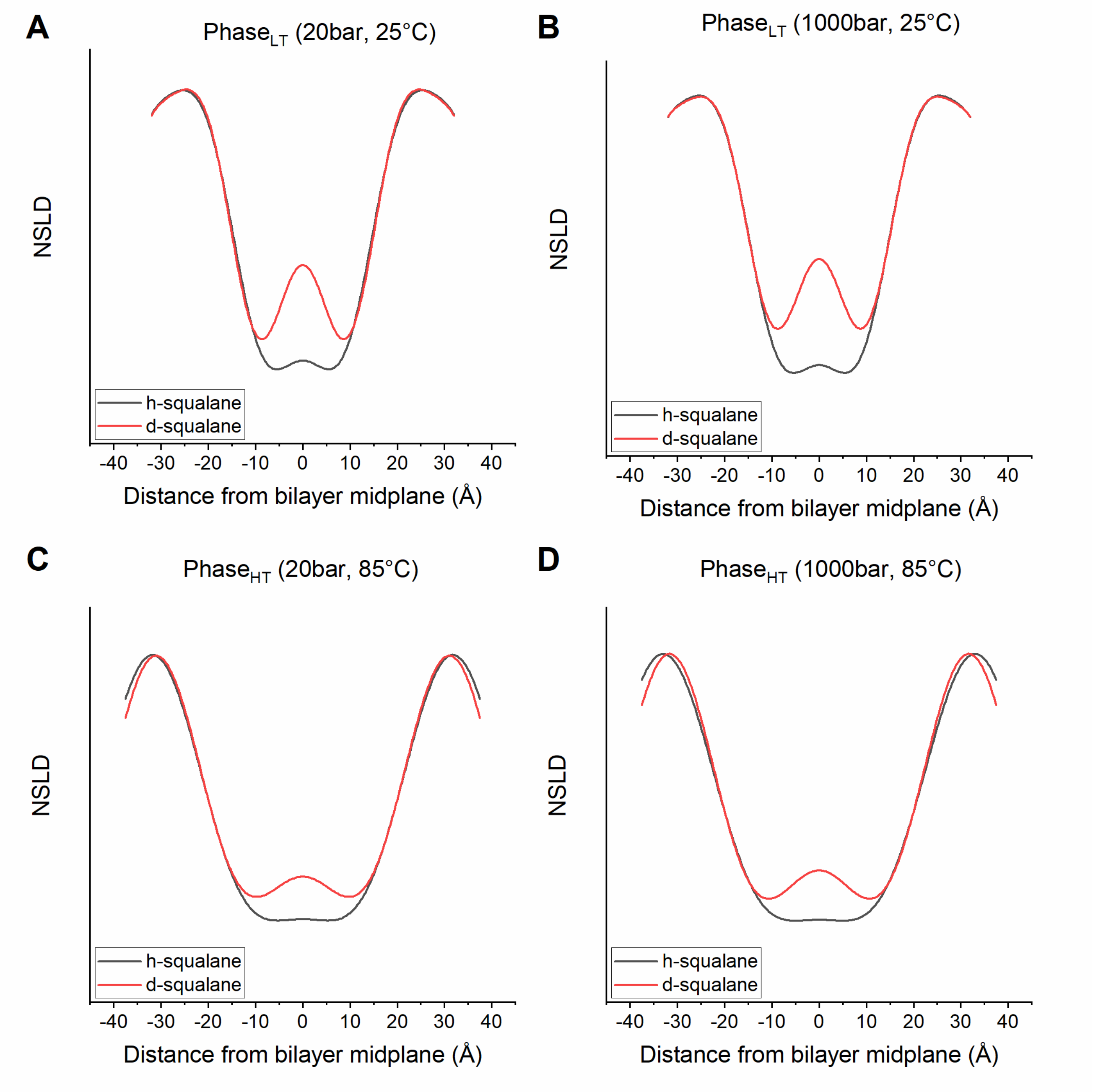

Supplementary Figure 2. Neutron Scattering Length Density (NSLD) plots of DoPhPC:DoPhPE (9:1) + 5 mol% hydrogenated-squalane (black) or deuterated-squalane (red). Phase$_{LT}$ at 25°C and a pressure of 20 bar **(A)** or 1000 bar **(B)**. Phase$_{HT}$ at 85°C and a pressure of 20 bar **(C)** or 1000 bar **(D)**.

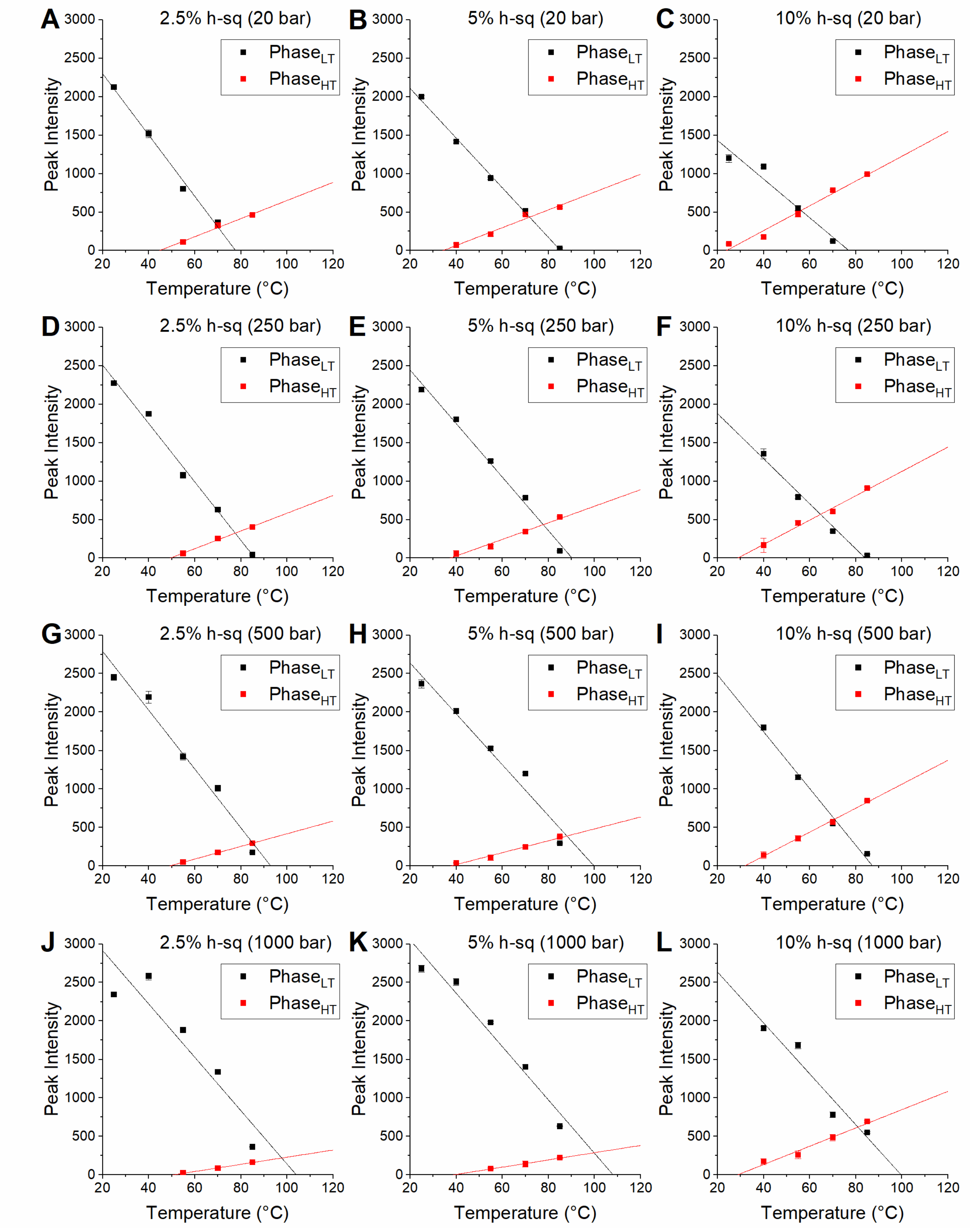

Supplementary Figure 3. Integrated intensity of first-order diffraction peak as a function of temperature for Phase$_{HT}$ (red) and Phase$_{LT}$ (black) for various pressures: 20 bar **(A-C)**, 250 bar **(D-F)**, 500 bar **(G-I)** and 1000 bar **(J-L)** and various percentages of squalane: 2.5 mol% **(A,D,G,J)**, 5 mol% **(B,E,H,K)** and 10 mol% **(C,F,I,L)**. Lines indicate linear fit of data.

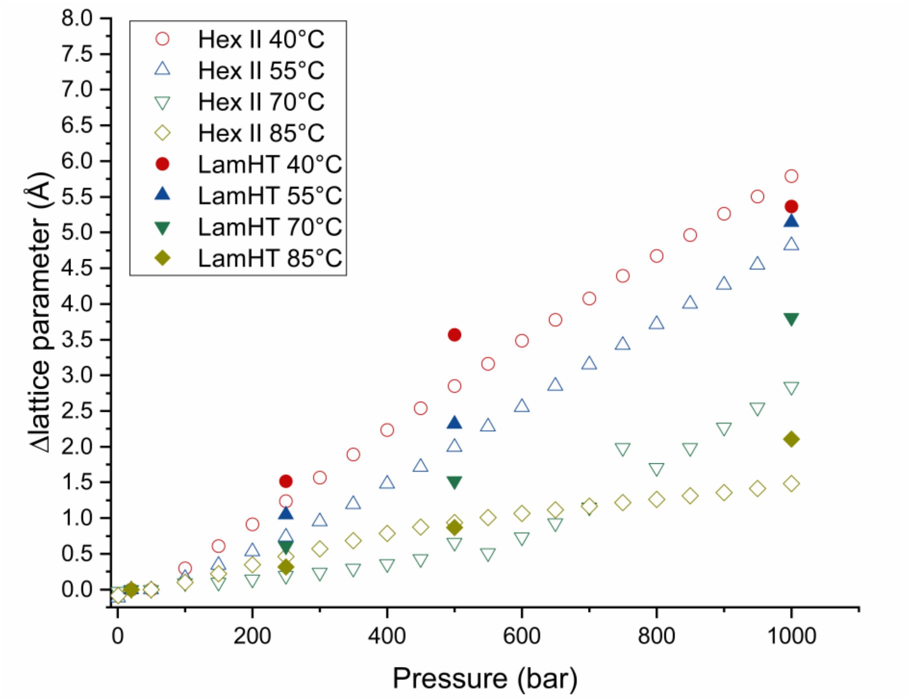

Supplementary Figure 4. Pressure induced swelling in DoPhPC:DoPhPE + 5 mol% squalane membrane. Open symbols: pressure induced swelling in the Hexagonal II phase seen by SAXS. Closed symbols: pressure induced swelling of lamellar Phase_{HT} seen by neutron diffraction. Temperature is 40°C (red), 55°C (blue), 70°C (green), and 85°C (yellow). Errors are ± 1 Å.

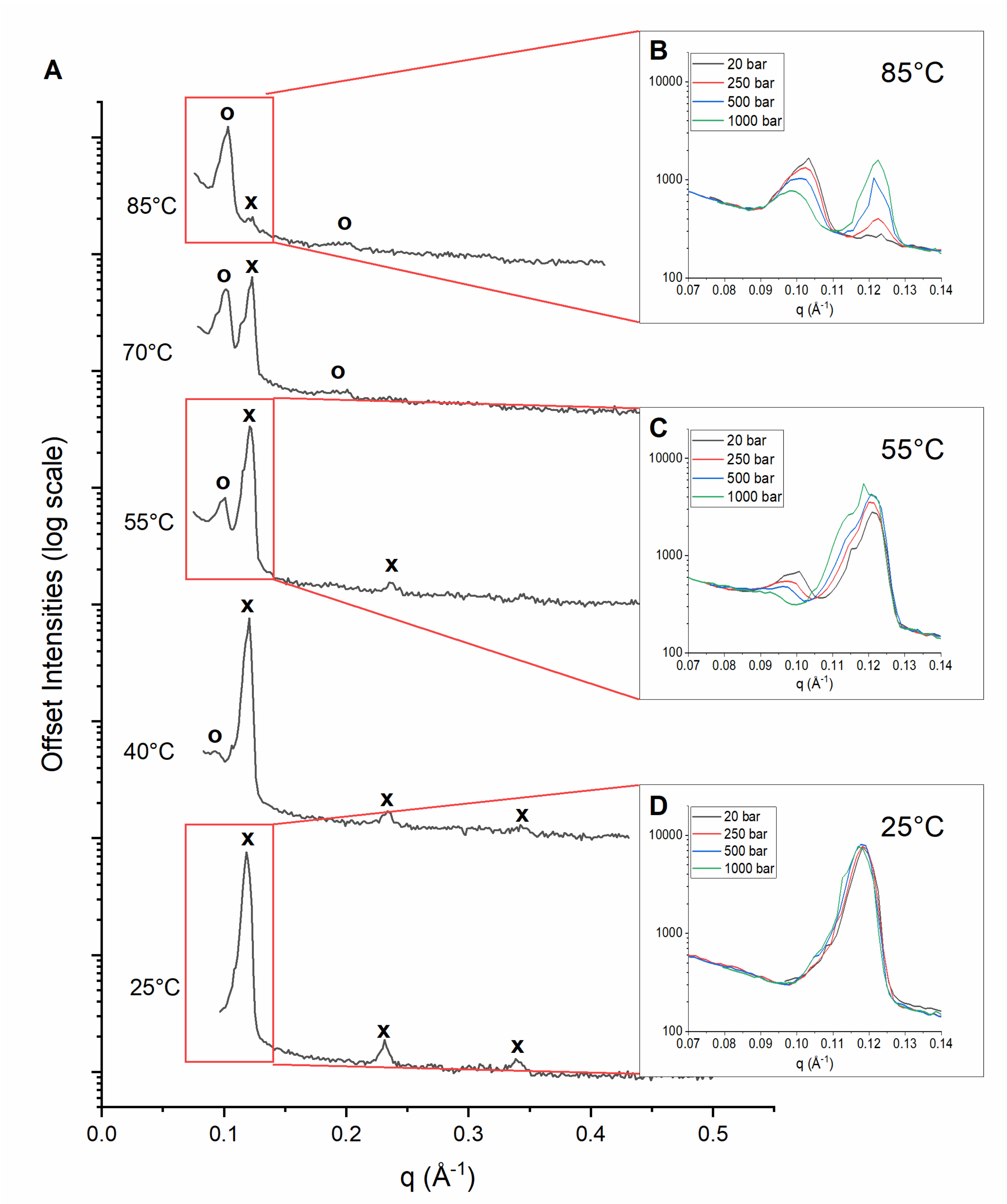

Supplementary Figure 5. 1D Neutron diffractograms of DoPhPC:DoPhPE (9:1) + 2.5 mol% squalane membrane at temperatures ranging from 25°C to 85°C. **(A)** Diffractograms at 20 bar. Diffraction peaks corresponding to Phase$_{LT}$ are denoted with an 'x' and peaks corresponding to Phase$_{HT}$ are denoted with an 'o'. First order diffraction peaks at 85°C **(B)**, 55°C **(C)** and 25°C **(D)** measured at 20 bar (black), 250 bar (red), 500 bar (blue) and 1000 bar (green).

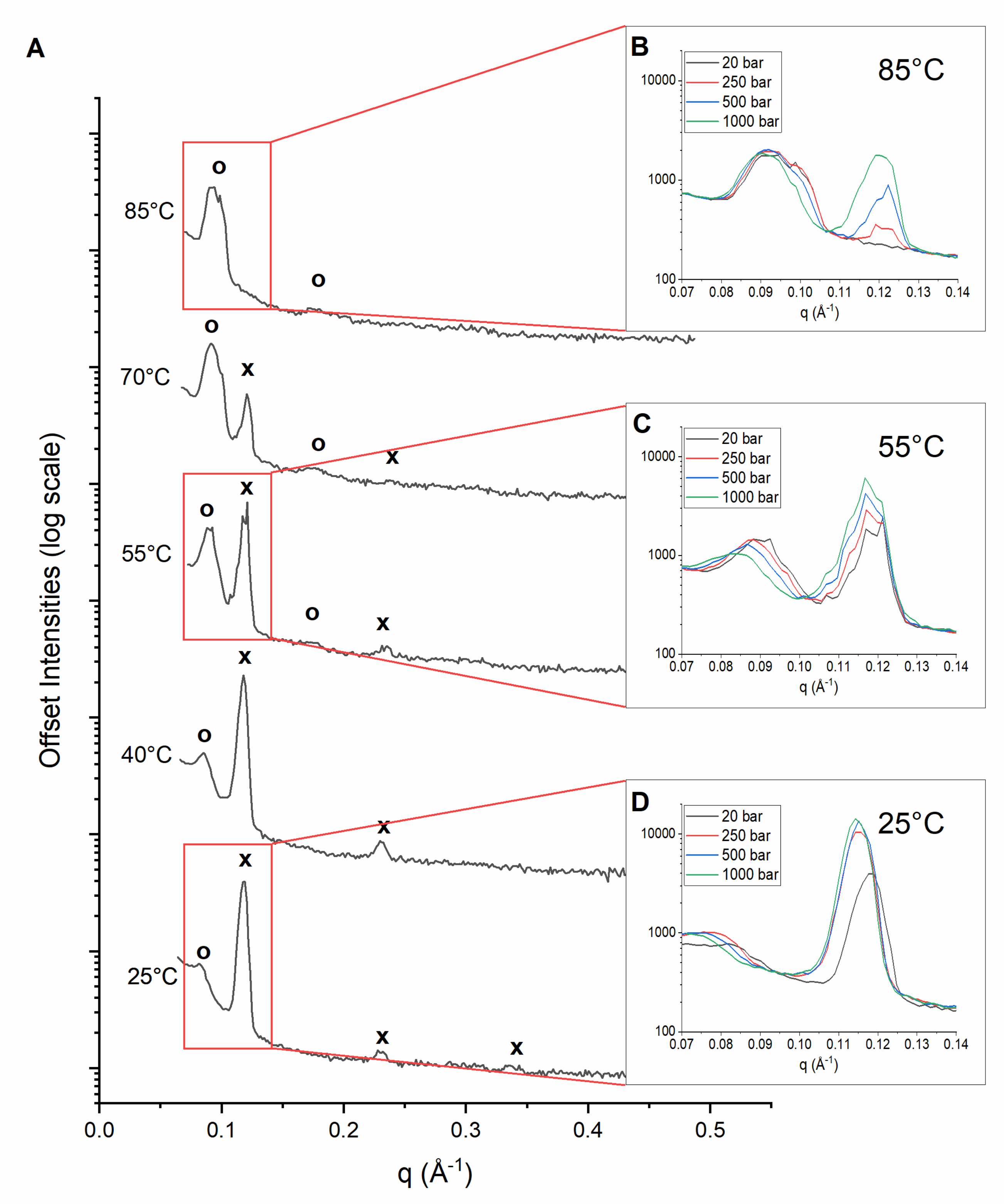

Supplementary Figure 6. 1D Neutron diffractograms of DoPhPC:DoPhPE (9:1) + 10 mol% squalane membrane at temperatures ranging from 25°C to 85°C. **(A)** Diffractograms at 20 bar. Diffraction peaks corresponding to $Phase_{LT}$ are denoted with an ‘x’ and peaks corresponding to $Phase_{HT}$ are denoted with an ‘o’. First order diffraction peaks at 85°C **(B)**, 55°C **(C)** and 25°C **(D)** measured at 20 bar (black), 250 bar (red), 500 bar (blue) and 1000 bar (green).

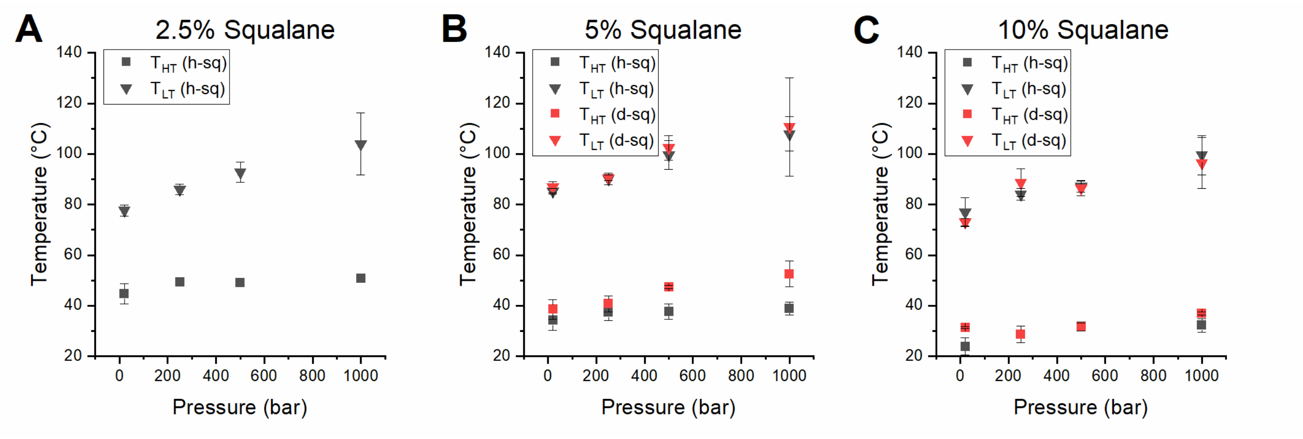

Supplementary Figure 7. Pressure/temperature phase diagrams for membranes containing 2.5 mol% squalane **(A)**, 5 mol% squalane **(B)** and 10 mol% squalane **(C)**. The temperature at which $Phase_{HT}$ appeared (T_{HT}) is shown in squares, and the temperature at which $Phase_{LT}$ disappeared (T_{LT}) is shown in triangles. Membranes containing hydrogenated squalane (h-sq) shown in black and deuterated squalane (d-sq) shown in red exhibited similar phase separation behavior.

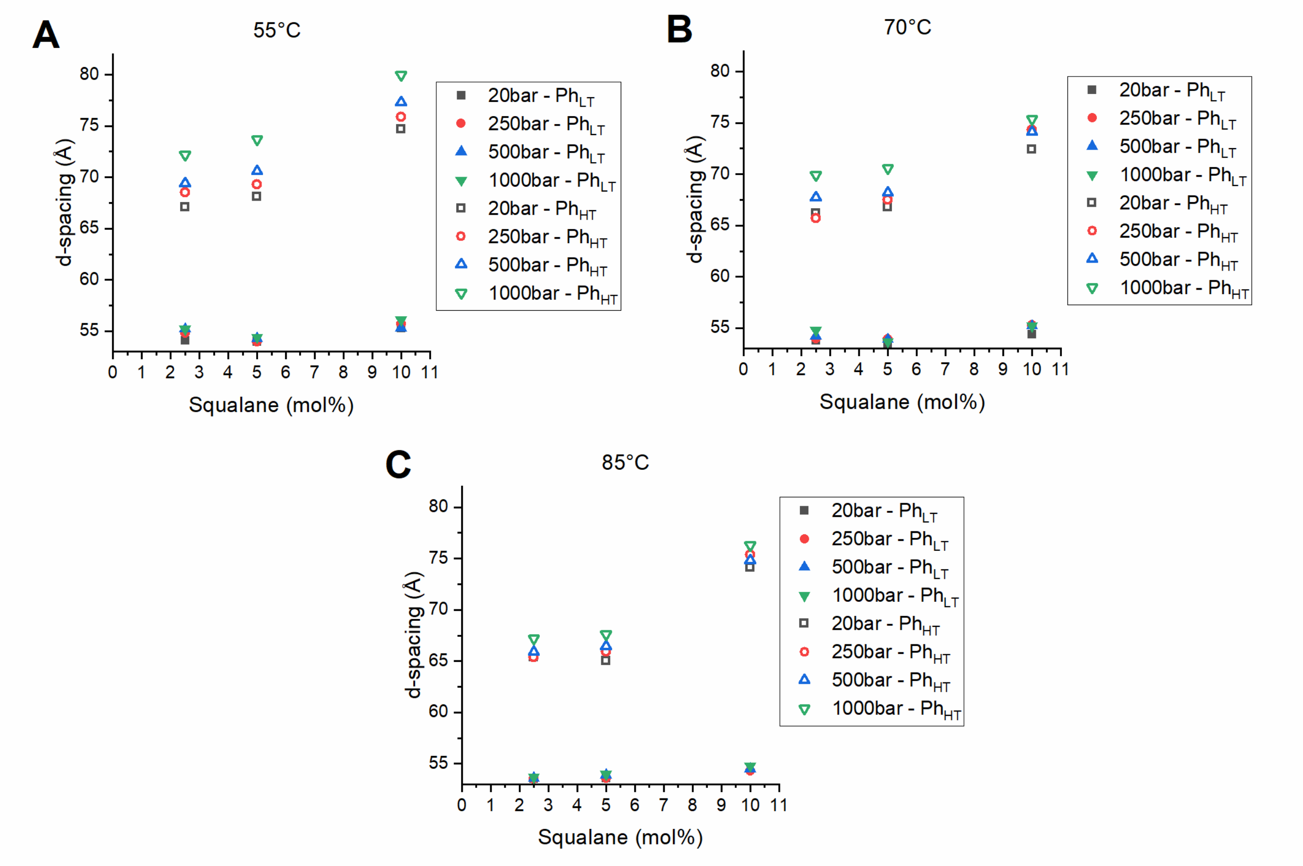

Supplementary Figure 8. Increase in membrane d-spacing as a function of squalane percentage. Temperature was 55 °C **(A)**, 70 °C **(B)** or 85 °C **(C)**. At these temperatures, both Phase$_{LT}$ (solid symbols) and Phase$_{HT}$ (open symbols) coexist for all squalane concentrations tested. The d-spacing was measured at 20 bar (black squares), 250 bar (red circles), 500 bar (blue triangles) or 1000 bar (green inverted triangles).
